# Wearable sensor-based spinal motion assessments for identifying phenotypic clusters in chronic low back pain

**DOI:** 10.1007/s00586-026-09781-9

**Published:** 2026-02-06

**Authors:** Prasath Mageswaran, Jonathan Dufour, Alexander Aurand, Ryan Gifford, Guy Brock, Yousef Alish, Gregory Knapik, Hamed Hani, Lindsay Hanes, Samantha Krening, Francesco Sammartino, Dukagjin Blakaj, Ehud Mendel, Melissa Tornero-Bold, Andrew Grossbach, Kristen Noon, Joanna Peng, Adam Farris, Varun Singh, Venkat Kavuri, Christina McGhee, Anthony Nguyen, Jaime Patterson, Whitney Luke, Nasir Hussain, Jayesh Vallabh, Tristan Weaver, William Marras

**Affiliations:** 1 Spine Research Institute, The Ohio State University, Columbus, United States; 2 Bioinformatics, The Ohio State University, Columbus, United States; 3 Wexner Medical Center, The Ohio State University, Columbus, United States; 4 Neurosurgery, Yale University, New Haven, United States

**Keywords:** Back pain, Spinal motion, Wearables, Function, Digital biomarker, Digital health, Objective metrics, Biopsychosocial, Clinical outcome assessment

## Abstract

**Purpose:**

Low back pain (LBP) is a debilitating condition with highly variable treatment responses due to its complex biopsychosocial nature. This study utilized standardized in-clinic sensor-based spinal motion assessments to identify distinct motion profile subgroups in LBP patients and examine their association with biopsychosocial measures and longitudinal outcomes after usual care.

**Methods:**

In this prospective observational study, 607 LBP patients underwent standardized sensor-based spinal motion assessments to quantify extent of low back impairment. Machine learning clustering of patient’s spinal motion signatures identified patient subgroups, which were compared across demographic, clinical, and biopsychosocial measures. Finally, we used patient’s global impression of change (PGIC) as our primary outcome to compare variation in 3-month responses between clusters after usual care treatments.

**Results:**

We identified three baseline motion-based clusters: Poor function (*n* = 179), Moderate function (*n* = 281), and High function (*n* = 147), reflecting low, moderate, and high spinal mobility. Demographic and clinical characteristics including age, body mass index, employment and comorbidity differed significantly across clusters. Notably, although clusters were derived using motion features only, we found significant variation in pain outcomes including pain intensity and interference. Moreover, cluster membership was independently associated with key biopsychosocial measures, including pain intensity, pain interference, physical function, social role, and self-efficacy, indicating that these phenotypes capture clinically relevant differences in pain experience and related biopsychosocial domains. At 3 months, PGIC responses differed significantly by cluster, with the Poor function group showing a higher proportion of negative outcomes.

**Conclusions:**

Three distinct motion-based clusters were identified that demonstrated variability across demographic, clinical, and biopsychosocial measures. Although derived solely from spinal motion, these clusters were associated with key patient-reported outcomes, suggesting that motion-based clustering may offer complementary insight into patient heterogeneity and identifying subgroups at risk of suboptimal outcomes.

**Clinical Trials Registration ID:**

NCT05776771.

**Key findings:**

**Question** Can sensor-enabled motion assessments be utilized for patient phenotyping to identify unique low back pain subgroups and predict treatment responders?

**Findings:**

Three distinct functional clusters were identified using wearable sensor-enabled motion assessments and cluster membership was significantly associated with 3-month responses.

**Meaning:**

The use of sensor-enabled motion assessments can inform patient heterogeneity and identify clusters associated with poor outcomes.

## Introduction

Low Back Pain (LBP) is the leading cause of long-term disability with over 540 million people affected globally [1–8]. It is estimated that up to 80% of the population will experience an episode of LBP at some point in their lives [9–11], and ~ 80–90% of all LBP cases are non-specific, meaning no identifiable source or cause of pain can be found [12]. LBP is also the most common condition for which opioids are prescribed, potentially contributing to the opioid crisis [13–16]. The medical costs associated with the treatment of LBP exceed $134 billion and are expected to rise with the aging population [6, 7]. Despite the increased use of various interventions and imaging technologies, the prevalence of LBP remains high, with no significant improvement in outcomes [17–19]. As a result, finding the most optimal treatment for individual patients suffering from LBP remains a challenging and resource-intensive task for healthcare providers.

This issue persists partly from the multifaceted and heterogeneous nature of the condition. LBP is not a singular condition; rather, it represents a complex interplay of physical, mental, and social factors that influence its onset, progression, evolution, and resolution. These complex biopsychosocial interactions that result in heterogenous manifestations of LBP pose a significant diagnostic and treatment challenge for clinicians. Additionally, the lack of objective metrics makes it challenging to reliably characterize a patient’s condition and benchmark disease severity, which further complicates clinical decision-making and often results in generalized treatment approaches for a diverse patient population instead of personalized care. Therefore, gaining a deeper understanding of these complex interactions by leveraging objective, quantifiable metrics to identify meaningful clinical phenotypes has the potential to enhance clinical decision-making, tailor and refine treatment strategies for an individual, and ultimately improve patient outcomes.

Clinically, pain and function are universally important and interconnected phenomena that impact patient outcomes. It is well known that pain can lead to subtle changes in motor control patterns, neuromuscular dysfunction and guarding behaviors [20, 21], all of which may be observable in the characteristics of spinal motion (motion signature). In our previous work, we demonstrated the novel application of wearable sensor-enabled functional spine assessments to objectively assess function impairment [21, 22]. This work highlighted strong associations between spinal motion features and pain-related function, as measured using validated instruments such as PROMIS Physical function or Oswestry Disability Index (ODI) [21–24]. Central to this approach are algorithms that compare a patient’s dynamic spinal movements during a standardized sensor-based spine motion test to a proprietary database of healthy controls (who completed the same motion test) to objectively characterize the extent of low back impairment [22, 23, 25–27]. These efforts have demonstrated that spinal motion signatures can reliably distinguish LBP patients from healthy controls [21, 22, 26], with high accuracy (AUC > 85%) and strong inter/intra-rater reliability (ICC > 0.8) [22, 28, 29]. This sensor-based motion assessment is grounded on the principle that movement is integral to daily activities of living and human function. By capturing how individuals move their spine, it aims to provide insights on how LBP affects spine function, reveal distinct phenotypic patterns and help guide more tailored treatment strategies to maximize clinical benefit.

The primary objective of this study was to identify low back pain phenotypes or clusters through unsupervised machine learning applied to spinal motion data collected via a novel wearable sensor-based motion assessment. Secondarily, we examined how these motion-derived phenotypes relate to demographic, clinical, and self-reported biopsychosocial measures. Exploratory analyses also assessed the association between cluster membership and 3-month Patient Global Impression of Change (PGIC) measure after usual care.

## Methods

### Study design and population

All study procedures (IRB protocol #: 2020H0250; Clinical Trials Registration #: NCT05776771) were approved by the Ohio State University’s Institutional Review Board (IRB) and all participants provided written informed consent prior to enrollment. This was an observational study designed to identify clusters or phenotypes in LBP using sensor-based spinal movement data. Participants with LBP (*n* = 607) were recruited based on eligibility criteria and medical history during care-seeking visits at partnering medical clinics in Columbus, Ohio. Upon enrollment after obtaining informed consent, participants completed a series of patient-reported outcome (PRO) questionnaires that target critical biopsychosocial domains and underwent a 6–8 min standardized functional motion assessment of the lumbothoracic (trunk) spine. All study assessments were conducted at baseline prior to usual care, and at 3-month follow-up after treatment.

### LBP patient population

Our participants were adults with LBP that sought a medical consult at the Ohio State University Wexner Medical Center (OSUWMC), Columbus, Ohio. The *inclusion criteria* were (a) age of 18 years or older; (b) currently seeking medical consult with primary complaint of low back pain; (c) able to stand for 10 min; and (d) able to speak, read and understand English. The *exclusion criteria* were: (a) known pregnancy; (b) current low back pain is the result of a traumatic injury or is positive for spine instability with imaging; (c) neoplasia including history of brain or spine cancer; (d) spinal deformity requiring medical treatment, (e) known spinal fractures in the last 6 months; (f) current osteomyelitis or spinal infection; (g) current condition requiring immobilization or bracing of the spine; (h) spinal fusion across 4 or more lumbar disc levels; (i) known osteoporosis; (j) current open wounds in the back; (k) known unstable spondylolisthesis; (l) blind or severe uncorrected vision problems that prevent participant from reading a tablet or laptop screen; (m) deaf or hearing problems that prevent the participant from hearing verbal instructions; (n) actively being treated by a medical provider for concussion; (o) has been diagnosed by a medical provider with severe vertigo, fainting, narcolepsy, or balance disorders with high risk for falling; and (p) any reason that a treating physician, researcher or participant determines it is unsafe to participate in the study.

### Study procedure

**Fig. 1:**
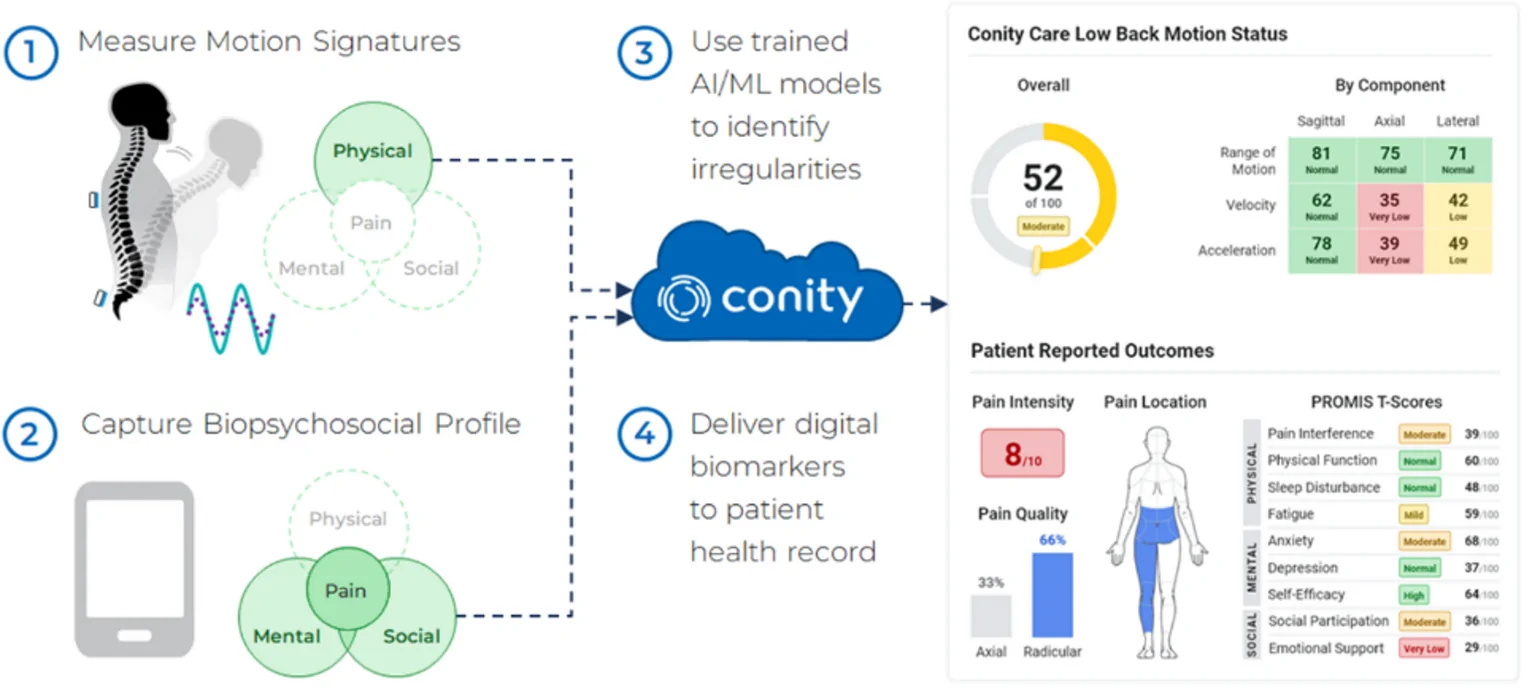
Functional Overview of Spine Health Platform showing motion assessment as well as biopsychosocial profiling to generate a comprehensive digital report (right)

Participants completed a series of PROs that encompassed key biopsychosocial domains (physical, mental and social), prior to performing a 6–8 min spinal motion assessment administered using a wearable sensor-based platform (Conity Care^™^) [21, 30]. Usual care treatments received between baseline and 3-month follow-up were also documented through a self-reported questionnaire. All data were collected at baseline and after 3-month follow-up using the Conity platform which is shown in Fig. 1. The platform enables a comprehensive assessment of spine health by integrating sensor-based motion evaluations and PROs along with intuitive summary reports for clinical inference.

### Sensor-based spinal motion assessment

**Fig. 2:**
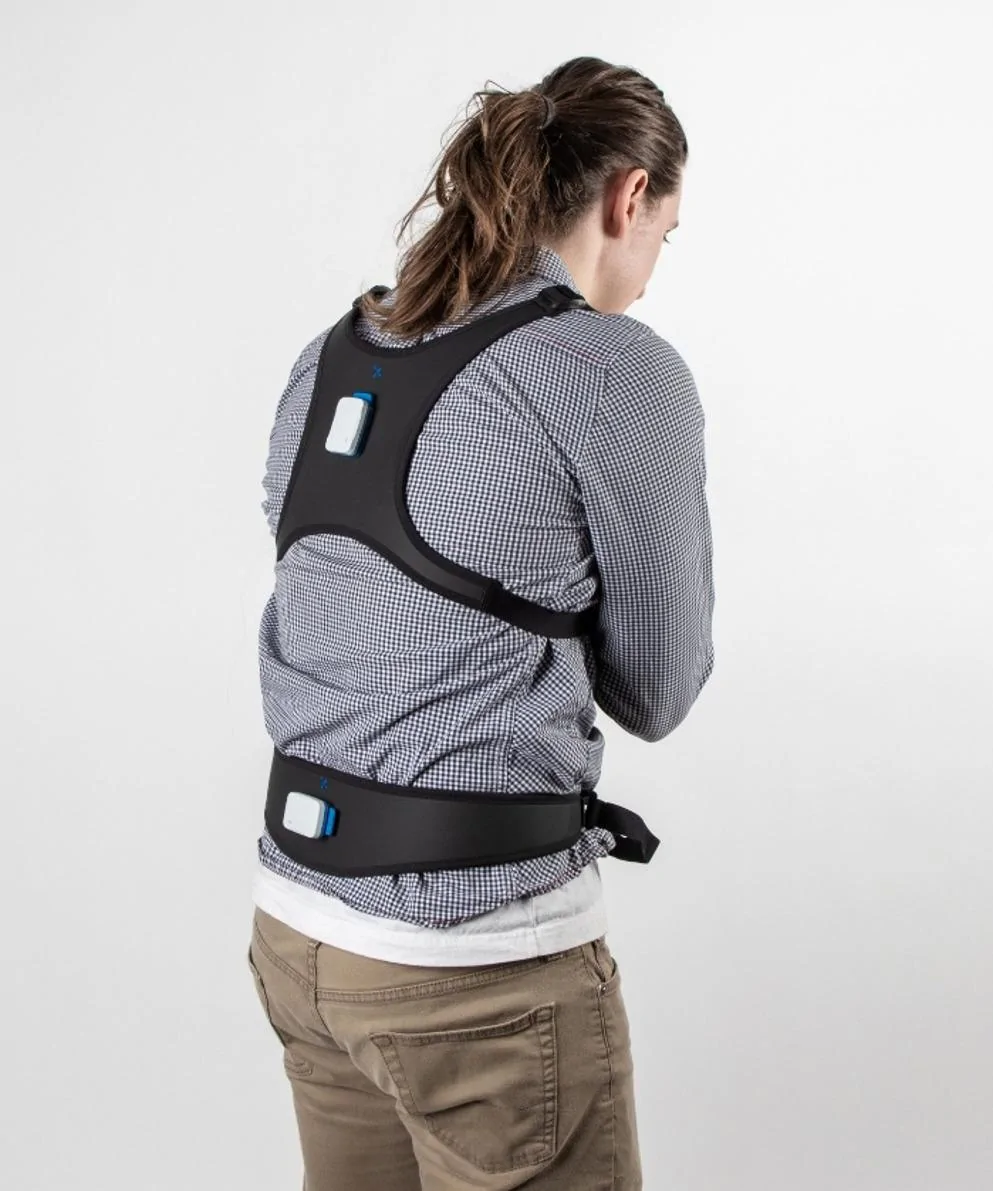
Back harness and sensor configuration for spinal motion assessment

The spine motion assessment is a standardized dynamic test designed to evaluate the 3D kinematic performance of the spine under controlled conditions. The entire test is structured to be completed in approximately 6–8 min. During the assessment, participants perform dynamic trunk movements in three anatomical planes: flexion and extension for sagittal spinal movements, right-to-left bending for lateral spinal movements, and right-to-left twisting for axial spinal movements. Each movement is performed under two distinct conditions or modes of movement. The first is a slow stretch, where participants complete a slow, controlled movement to assess maximal pain-limited range of motion. The second mode involves fast dynamic repetitive controlled movements to evaluate speed and control. Participants are instructed to move as far as they can safely for the slow stretch, whereas for the dynamic movement tasks, they were instructed to move as quickly and as far as they can comfortably. Custom inertial measurement unit (IMU) sensors, mounted on harnesses (Fig. 2), are worn on the chest and pelvis to quantify these spine-specific movements. The platform software includes embedded audio, video, and graphic instructions to guide both participants and evaluators through the data collection protocol [21]. All spinal motions are recorded directly into the Conity platform.

Collected motion data were automatically processed using proprietary algorithms to extract key motion features and made available for reporting immediately after collection [23]. A total of 9 motion features were extracted based on maximum value observed during repetitive cyclic movements. These included 3 rotational range of motion (ROM) measures derived from slow stretching movements, as well as 3 maximum velocities and 3 maximum accelerations derived from dynamic fast movements assessed across the axial, lateral, and sagittal planes. Finally, a composite metric (Motion Index) is generated using a discriminant modeling framework calibrated against a proprietary database of asymptomatic controls who completed the same test. This composite score provides an objective measure of functional impairment that captures deviations from healthy motion signatures for enhanced clinical interpretability [21–23, 30]. The score ranges between 0 and 100 with higher scores indicating healthier spinal function. Motion index scores that exceed 60 are generally comparable to the 50th percentile of healthy controls. It has demonstrated good to excellent intra- and inter-rater reliability in assessing spinal function [25].

### Study measures

The study utilized a comprehensive set of objective spinal motion features and subjective patient reported outcome measures (questionnaires) to identify, characterize and evaluate phenotypic clusters or subgroups. These measures (described below) were selected based on their well-established relevance and importance to LBP [31, 32]. Collectively, they enabled a more comprehensive biopsychosocial profiling of patients with LBP. Brief descriptions of these measures are presented below:

### Candidate spinal motion features used to *derive* the phenotypic clusters

#### Spinal motion features

Spinal motion features extracted from wearable sensor-enabled functional assessments were used as inputs to identify phenotypes or clusters. These 9 motion features encompass three-dimensional kinematic data that can be summarized by total ROM, as well as the maximum velocity and maximum acceleration ranges across the three anatomical planes: axial, lateral, and sagittal. By capturing the spine’s dynamic behavior in these planes, the data provides a comprehensive view of spinal motion, facilitating the identification of distinct movement patterns and their potential relationship to LBP experience.

### Candidate measures used to *evaluate* the phenotypic clusters

#### Demographic and clinical characteristics

Participant characteristics (individual and clinical) collected at baseline included age; race; ethnicity; body mass index (BMI); smoking status; medical history; education level; employment status; pain duration; marital status and comorbidities included headaches, hip pain, widespread joint pain, cardiovascular disease, vertigo, high blood pressure, diabetes, kidney, liver, Parkinson’s, Multiple sclerosis, psychiatric conditions.

#### Biomechanical Domain - Motion index

This is a composite score that summarizes the extent of functional impairment based on comparisons to a healthy control database [21, 22, 30]. It leverages three-dimensional measurements of rotational joint position, velocity, and acceleration extracted from the standardized sensor-enabled functional motion test with demonstrated classification accuracy of over 85% in distinguishing between healthy controls and patients with LBP. The score ranges between 0 and 100 with lower scores indicating poor function and higher scores indicating healthy function [22, 23]. It provides a uniquely objective and different dimension to a patient’s pain experience.

#### *Pain-related Domain* - pain Intensity, and pain interference

We assessed average pain intensity (over 7 days) using a PROMIS pain intensity scale of 0 (no pain) to 10 (worst pain imaginable). In addition, PROMIS pain interference domain was also assessed using PROMIS item banks [33–38]. These domains are scored on a T-score metric, with a mean of 50 normalized to the general US population and a standard deviation of 10. For PROMIS Pain interference, lower scores indicate less pain interference.

#### *Psychological Domain* - depression, and anxiety

Psychological factors such as depression and anxiety are known indicators that are linked with LBP and can impact patient outcomes [39–41]. In this study, depression, and anxiety were assessed using PROMIS item banks [42–44]. These domains are also scored on a T-score metric that ranges between 10 and 90 with a mean of 50 and standard deviation of 10 in a reference population. For PROMIS Depression and Anxiety, lower scores indicate less psychosocial distress.

#### *Pain impact Domain* - physical function, social Role, sleep disturbance and fatigue

Other factors of interest that were explored included physical function, social role (perceived ability to participate in one’s usual social roles and activities), sleep disturbance and fatigue, which are known to be impacted by LBP experience and can affect patient outcomes. We used PROMIS Physical Function, Social Role, Sleep Disturbances and Fatigue item banks to evaluate and document these factors [33, 35, 45]. For PROMIS Physical Function and Social Role, higher scores indicate higher physical and social functional states, while for PROMIS Sleep Disturbance and Fatigue, lower scores indicate less sleep disturbance and lower levels of fatigue.

#### *Behavioral Domain* - fear avoidance behavior, pain catastrophizing and Self-efficacy

A 4-item Fear-Avoidance Behavior Questionnaire (FABQ) was used to characterize fear avoidance beliefs related to physical activity [46]. Higher scores indicated pain-related fear avoidance behavior. In addition, a 6-item Pain Catastrophizing Scale (PCS) was used to assess catastrophizing behaviors in relation to LBP [47, 48]. Lastly, we administered the 4-item PROMIS Self-efficacy questionnaire to assess a patient’s belief in their ability to manage their health condition, with higher T-scores reflecting greater self-efficacy [49].

#### *Primary Outcome* - patient’s global impression of change (PGIC)

We assessed longitudinal outcomes at 3-month follow-up using PGIC [50] questionnaire to assess patient’s overall impression of their well-being after usual care. The PGIC ratings of “Very much improved”, “Much improved”, and “Minimally improved” were used to identify responders within each cluster, while non-responders were those patients that indicated the following: “Very much worse”, “Much worse”, and “Minimally worse”.

### Statistical analysis

Our analytical approach was performed as follows: First, high-dimensional dynamic motion features (velocity and acceleration features) were transformed using square root and cube root functions, respectively, to achieve a normal distribution. Participants with missing or incomplete motion data at baseline were excluded. Additionally, given the variation in scales, all continuous variables were standardized to z-scores using the standard scaler from the Scikit-learn library [51] to ensure comparability across features. Second, we performed a Principal Component Analysis (PCA) to reduce the dimensionality of the motion feature dataset, prior to clustering. This is particularly needed as many of the features are highly correlated. Third, we applied an unsupervised K-means clustering algorithm [52] leveraging the PCA components to identify distinct low back pain (LBP) clusters, grouping participants with similar baseline motion characteristics. The optimal number of clusters was determined by the “elbow criterion,” which assesses within-cluster variance to pinpoint where additional clusters cease to significantly enhance model performance. Furthermore, descriptive statistics were utilized to summarize the clusters, with categorical variables presented as frequencies and percentages, and continuous variables expressed as means with their corresponding standard deviations. In addition, Chi-Square or Fisher’s Exact test for categorical variables, or one-way Analysis of Variance (ANOVA) with Tukey-Kramer multiple comparison test for continuous variables was conducted to evaluate mean differences between individual clusters. Finally, we used logistic regression to assess association between clusters and longitudinal outcome at 3-month follow-up after usual care. All statistical analyses were conducted using Python and Scikit-learn libraries. P-value < 0.05 was considered statistically significant.

## Results

### Demographic and clinical characteristics

**Fig. 3:**
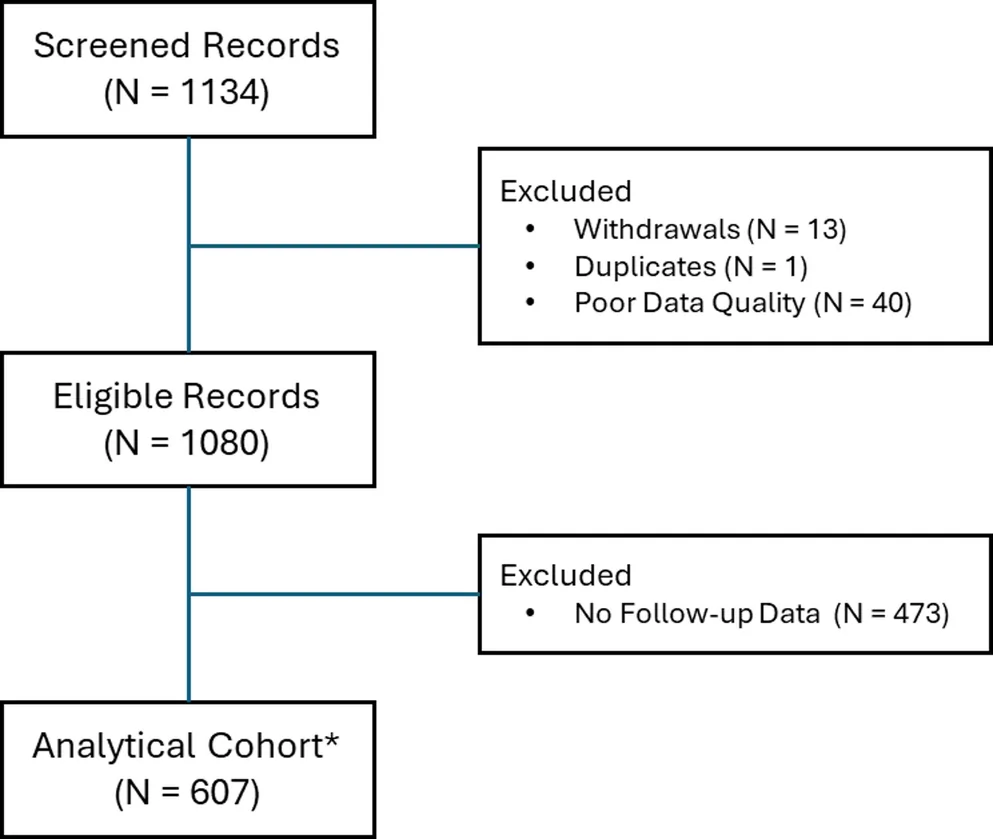
Participant flow diagram from eligibility screening to inclusion for analysis. * Includes 3-month follow-up data on primary outcome, PGIC

Our final analytical cohort with baseline and 3-month follow-up data consisted of 607 LBP patients (65% females; mean age: 51.6 ± 14.9 years; mean BMI: 32.2 ± 7.9). Figure 3 shows the participant flow diagram highlighting eligibility screening and inclusion into the analytical cohort. Between baseline and the 3-month follow-up, the most common treatments prescribed based on self-reports were physical therapy (73%), medications (72%), complementary and integrative medicine (55%), psychological health interventions (18%), interventional procedures (28%), and surgery (5%). Among medications, 61% received NSAIDs, 29% anticonvulsants, 16% SSRIs or SNRIs, 11% opioids, and 7% tricyclic antidepressants. Physical therapy (PT) included active PT (48%), passive PT (14%) and exercise (60%). Complementary treatments included spinal manipulation or chiropractic care (16%), mindfulness practice (33%), acupuncture (6%), and dietary interventions (39%). Psychological health interventions were mostly related to mental health evaluations (19%). Interventional procedures included injections (25%), radiofrequency ablation (3%), and spinal cord stimulation (2%). Finally, surgical interventions included fusion surgery (1%) and other lumbar procedures (5%).

### Dimensionality reduction and clustering analysis

**Fig. 4:**
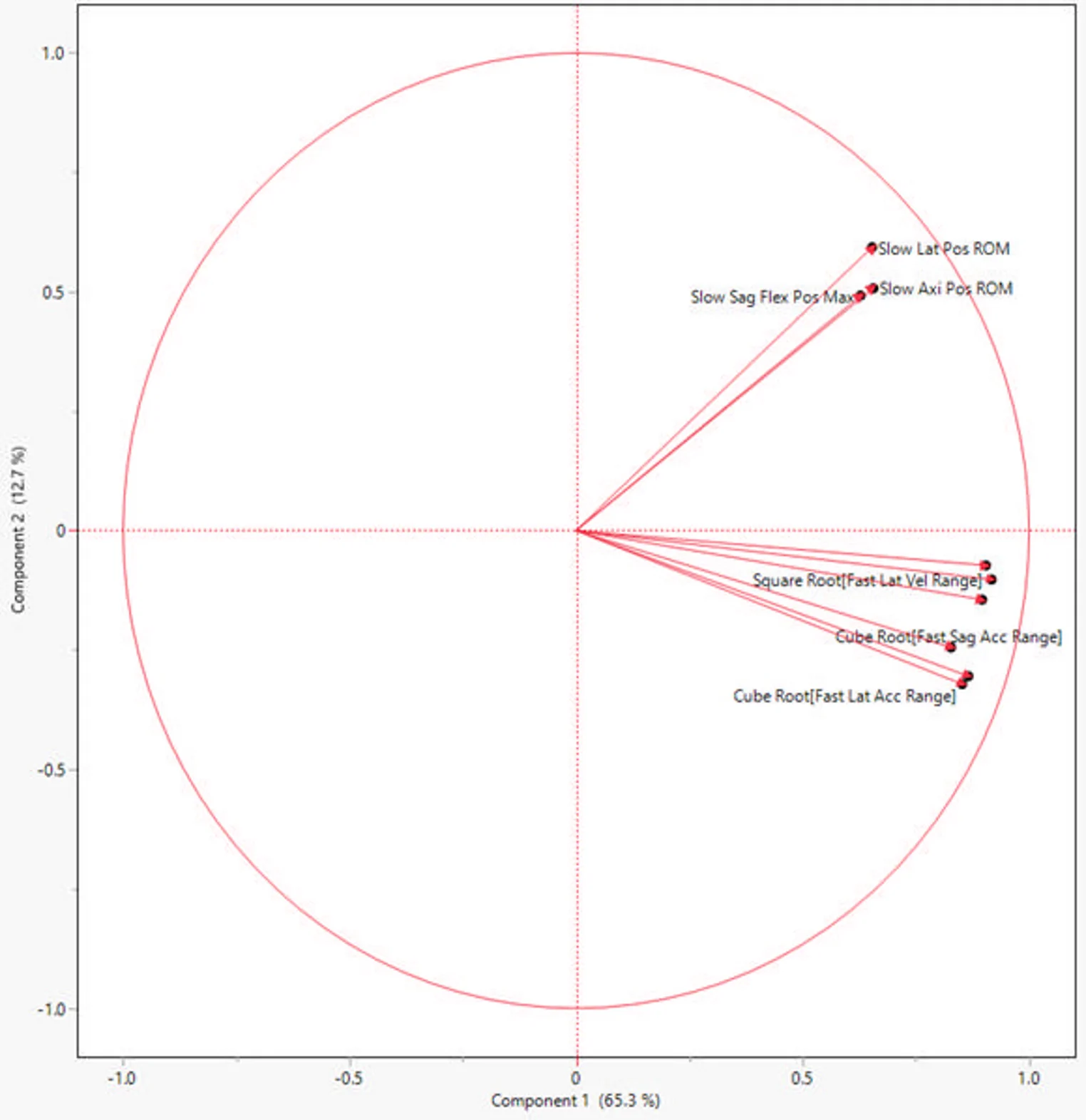
Biplot showing loadings of the kinematic features along with variance explained by each principal component. Component 1 showed positive loadings of ROM, velocities and acceleration features while Component 2 were primarily positive loading of ROM features and negative loadings of velocities and acceleration features across axial, lateral and sagittal planes

Our PCA identified two dominant patterns in motion feature data that together explained 78% of the total variance in the data. The first pattern (Component 1) reflected overall spinal movement capacity, with high values driven by higher ROM, higher velocities and higher acceleration features. The second pattern (Component 2) reflected a contrasting movement profile characterized by higher ROM features coupled with slower dynamic movements, as indicated by lower velocity and acceleration features. Figure 4 shows the biplot for the two dominant patterns and the contribution of each motion feature.

**Fig. 5:**
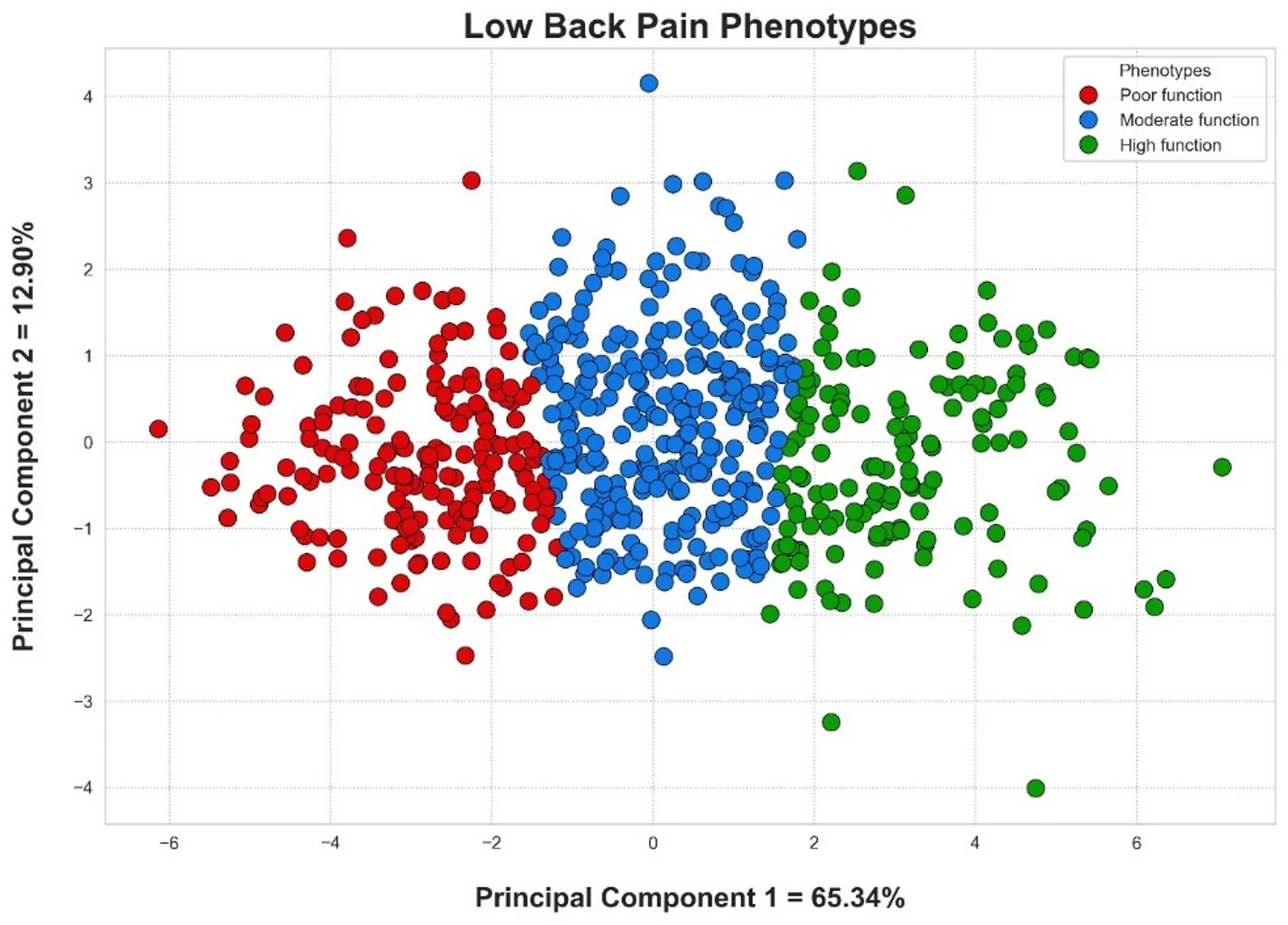
Three distinct low back pain clusters were determined indicating variation in spinal movement patterns

**Fig. 6:**
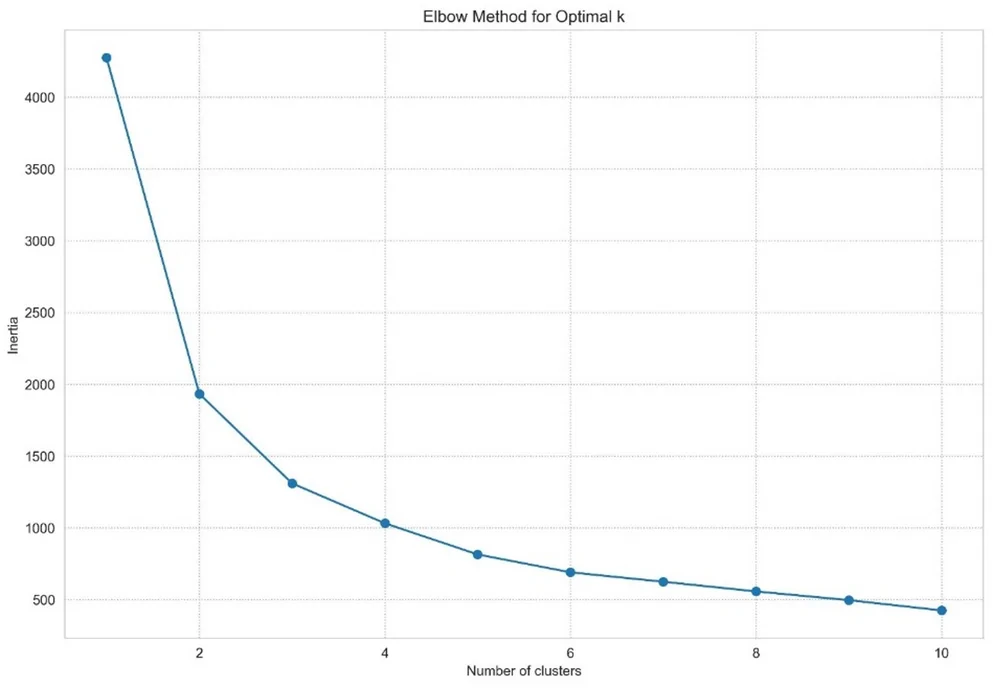
Optimal clusters were determined using elbow method

Our clustering analysis using motion features (ROM, velocities and accelerations) identified 3 distinct LBP clusters (Fig. 5, ordered from poor function to high function) with a Silhouette Score of 0.38. Optimal number of clusters were selected using the elbow criterion (Fig. 6). Table 1 summarizes the motion characteristics of each cluster. The clusters (labeled Poor, Moderate, and High function) demonstrated significant differences in spinal motion (*p* < 0.0001), with motion values progressively increasing from Poor to High function cluster. Motion values in the High function cluster were closely similar to those observed in healthy individuals (Motion index scores that exceed 60 are generally comparable to healthy controls). Patients in the Poor function cluster also exhibited generally slower movements relative to other clusters.

**Table 1:** Variation in motion features between clusters

Measures	Poor function Mean (SD) *n* = 179	Moderate function Mean (SD) *n* = 281	High function Mean (SD) *n* = 147	*P-value	
**Axial**					
ROM (degrees)	38.2 (11.2) ^a^	55.6 (12.5) ^b^	63.9 (11.6) ^c^	< 0.0001**	
Velocity (degrees/sec)	98.8 (33.7) ^a^	179.1 (45.2) ^b^	296.3 (74.9) ^c^	< 0.0001**	
Acceleration (degrees/Sect. ^2^)	581.9 (221.6) ^a^	1048.5 (354.4) ^b^	2007.4 (693.1) ^c^	< 0.0001**	
**Lateral**					
ROM (degrees)	36.0 (10.7) ^a^	51.1 (12.2) ^b^	58.6 (13.5) ^c^	< 0.0001**	
Velocity (degrees/sec)	76.1 (22.7) ^a^	127.6 (27.6) ^b^	198.1 (46.9) ^c^	< 0.0001**	
Acceleration (degrees/Sect. ^2^)	339.2 (134.3) ^a^	571.9 (179.3) ^b^	1027.3 (366.6) ^c^	< 0.0001**	
**Sagittal**					
ROM (degrees)	24.8 (9.1) ^a^	37.5 (12.0) ^b^	47.0 (13.9) ^c^	< 0.0001**	
Velocity (degrees/sec)	80.9 (26.6) ^a^	140.3 (36.7) ^b^	231.6 (56.8) ^c^	< 0.0001**	
Acceleration (degrees/Sect. ^2^)	546.7 (257.5) ^a^	960.9 (375.9) ^b^	1737.9 (608.3) ^c^	< 0.0001**	

SD, Standard Deviation; Raw values of velocity and acceleration features are presented in the table above; *P-values are for comparisons across multiple clusters, with *p* < 0.05 indicating statistically significant differences between the 3 clusters; Letters (a, b, and c) indicate clusters that are significantly different from each other based on multiple comparisons; ** Significant differences across all three clusters based on multiple comparison

Demographic and clinical characteristics were compared across the three clusters, as summarized in Table 2. No significant differences (*p* > 0.05) were observed for birth sex, marital status, smoking status, pain duration, or radicular symptoms. Most participants in all clusters were non-smokers and married or in domestic partnerships. However, significant differences were found in age (*p* < 0.001) and BMI (*p* < 0.001), with the Poor function cluster comprising older individuals with higher BMI. Employment status also differed significantly (*p* = 0.001), with the Poor function cluster having the highest proportion of unemployed participants. Among self-reported comorbidities, widespread joint pain (*p* = 0.0099), high blood pressure (*p* < 0.0001), and diabetes (*p* = 0.037) showed significant variation across clusters while no significant differences we observed for neck pain (*p* = 0.36) and hip pain (*p* = 0.54).

**Table 2:** Demographic and clinical characteristics between motion-based clusters

Measures	Poor function Mean (SD) Count (%) *n* = 179	Moderate function Mean (SD) Count (%) *n* = 281	High function Mean (SD) Count (%) *n* = 147	*P-value
**Age**	56.5 (13.9) ^a^	50.9 (14.5) ^b^	46.8 (15.1) ^c^	< 0.001**
Birth Sex				0.64
Female	112 (63%)	188 (67%)	96 (65%)	
Male	67 (37%)	93 (33%)	51 (35%)	
**BMI**	35.1 (7.6) ^a^	31.9 (7.9) ^b^	29.3 (7.1) ^c^	< 0.001**
Marital Status				0.37
Single/Divorced/Separated/ Widowed/Never married	71 (40%)	96 (35%)	52 (37%)	
Married/Domestic partnership	108 (60%)	183 (65%)	95 (63%)	
**Employment Status**				0.001**
Employed	82 (49.9%) ^a^	167 (64%) ^b^	101 (72%) ^c^	
Not Employed	83 (50.1%) ^a^	94 (36%) ^b^	40 (28%) ^c^	
Non-Smokers	133 (84%)	209 (84%)	113 (83%)	0.98
Pain Duration				0.75
Less than 3 months	8 (5%)	8 (3%)	6 (4%)	
3–6 months	15 (9%)	16 (6%)	15 (11%)	
6–12 months	14 (8%)	24 (9%)	15 (11%)	
1–5 years	48 (29%)	83 (32%)	41 (29%)	
More than 5 years	81 (49%)	130 (50%)	61 (44%)	
Radicular symptoms				
Below Knee	65 (40%)	103 (40%)	44 (32%)	0.43
Buttock or Thigh	116 (71%)	192 (74%)	92 (67%)	0.64
**Comorbidities**				
Hip pain	84 (47%)	133 (48%)	62 (42%)	0.54
**Widespread joint pain**	84 (47%) ^a^	112 (40%) ^b^	45 (31%) ^c^	0.0099**
**High blood pressure**	84 (47%) ^a^	96 (34%) ^b^	35 (24%) c	< 0.0001**
Neck pain	66 (37%)	116 (41%)	51 (35%)	0.36
**Diabetes**	36 (20%) ^a^	37 (13%) ^b^	16 (10%) ^c^	0.037**

SD, Standard Deviation; * P-values are for comparisons between clusters with *p* < 0.05 indicating significant differences. Letters (a, b, and c) indicate clusters that are significantly different from each other based on multiple comparisons; ** Significant differences across all three clusters based on multiple comparison

The **poor function cluster** (*n* = 179) was characterized by older individuals (mean age: 56.5 ± 13.9 years) with the highest average BMI (35.1 ± 7.6), moderate to high levels of pain intensity (6.0 ± 2.3), and markedly impaired spinal mobility (Motion Index: 14.2 ± 4.9), indicative of significant functional limitation or restriction to spinal function. This group also reported a high prevalence of comorbidities, including hip pain (47%), widespread joint pain (47%), high blood pressure (47%), neck pain (37%), and diabetes (20%).

The **moderate function cluster** (*n* = 281), representing the largest subgroup, consisted of overweight individuals (BMI: 31.9 ± 7.9) with moderate to high pain intensity (5.5 ± 2.2) and intermediate spinal function (Motion Index: 35.3 ± 9.4). Common comorbidities reported in this cluster included hip pain (48%), widespread joint pain (40%), high blood pressure (34%), neck pain (41%), and diabetes (13%).

The **high function cluster** (*n* = 147) included younger participants (mean age: 46.8 ± 15.1 years) with slightly elevated BMI (29.3 ± 7.1), low to mild pain intensity (4.7 ± 2.4), and high spinal mobility (Motion Index: 61.4 ± 12.1), reflective of better functional status. Reported comorbidities in this cluster were less frequent and included hip pain (42%), widespread joint pain (31%), high blood pressure (24%), neck pain (35%), and diabetes (10%).

### Association with biopsychosocial measures

Cluster membership demonstrated significant association with multiple biopsychosocial measures. One-way ANOVA revealed statistically significant differences across clusters for a wide array of pain-related biopsychosocial measures (Table 3), including pain intensity (*p* < 0.0001), motion index (*p* < 0.0001), pain interference (*p* < 0.0001), physical function (*p* < 0.0001), social role participation (*p* = 0.0003), and self-efficacy (*p* < 0.0001). Conversely, no significant cluster differences were observed for pain catastrophizing (*p* = 0.15), anxiety (*p* = 0.54), depression (*p* = 0.15), fatigue (*p* = 0.05), sleep disturbance (*p* = 0.25), or fear avoidance (*p* = 0.16). Post hoc multiple comparisons further confirmed that pain intensity, pain interference, physical function, social role participation, self-efficacy, and motion index significantly differed across all three clusters. Finally, in exploring usual care treatments, no significant differences were found in the different types of treatments received by each cluster.

**Table 3:** Variation in pain-related biopsychosocial measures between motion-based clusters

Measures	Poor function Mean (SD) *n* = 179	Moderate function Mean (SD) *n* = 281	High function Mean (SD) *n* = 147	*P-value
Pain Intensity	6.0 (2.3) ^a^	5.5 (2.2) ^b^	4.7 (2.4) ^c^	< 0.0001**
Motion Index	14.2 (4.9) ^a^	35.3 (9.4) ^b^	61.4 (12.1) ^c^	< 0.0001**
Pain Interference	65.7 (6.9) ^a^	64.0 (6.7) ^b^	61.6 (7.8) ^c^	< 0.0001**
Physical Function	36.3 (5.5) ^a^	38.6 (5.6) ^b^	40.8 (6.3) ^c^	< 0.0001**
Social Role	41.5 (7.9) ^a^	44.3 (8.0) ^b^	46.3 (8.9) ^c^	< 0.0001**
Anxiety	53.5 (10.5)	53.7 (9.7)	52.5 (10.5)	0.54
Depression	51.2 (9.4)	50.8 (9.7)	49.2 (9.0)	0.15
Sleep Disturbance	57.5 (9.1)	56.1 (9.1)	56.1 (8.8)	0.25
Fatigue	58.2 (10.4)	56.3 (10.1)	55.5 (10.8)	0.05
Self-Efficacy	42.0 (6.5) ^a^	43.3 (7.2) ^b^	45.3 (8.5) ^c^	0.0003**
Pain Catastrophizing	9.8 (5.1)	9.3 (5.3)	8.6 (5.4)	0.15
Fear Avoidance	15.7 (5.8)	15.4 (5.7)	14.5 (5.8)	0.16

SD, Standard Deviation; * P-values are for multiple comparisons between clusters with *p* < 0.05 indicating statistical difference; Letters (a, b, and c) indicate clusters that are significantly different from other clusters based on multiple comparisons; ** Significant differences across all three clusters based on multiple comparisons. PROMIS Physical Function, and Social Role, higher scores indicate better health, while for the other PROMIS domains (Pain intensity, Pain Interference, Anxiety, Depression, Fatigue and Sleep disturbance) lower scores indicate better health

**Fig. 7:**
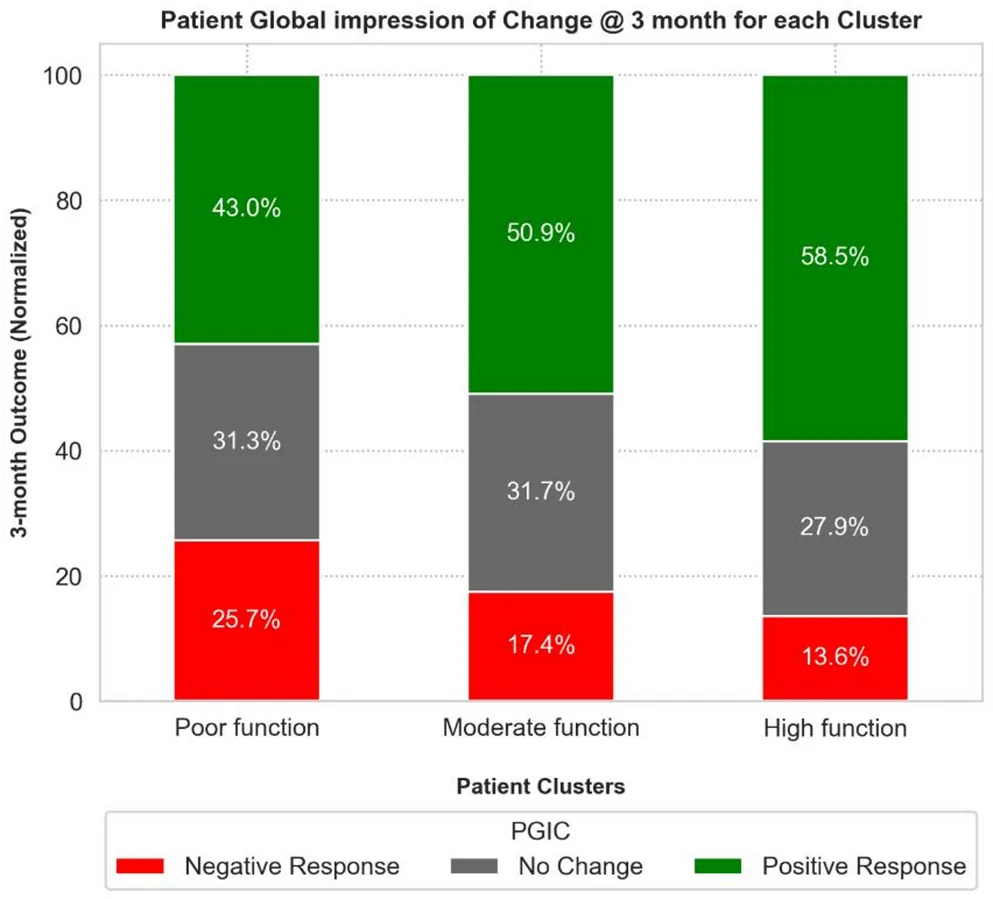
Variation in PGIC responses between clusters at 3-month follow-up with only ~ 43% of patients in the Poor function cluster indicating positive outcome compared with 50.9% and 58.5% of patients in Moderate function and High function cluster respectively

Explorative analysis of 3-month PGIC responses (Fig. 7) revealed that 43% (*n* = 77) of patients in the Poor function cluster reported a positive outcome, compared to 50.9% (*n* = 143) in the Moderate and 58.5% (*n* = 86) in the High function clusters. In contrast, 25.7% (*n* = 46) of patients in the Poor function cluster reported a negative outcome, compared to 17.4% (*n* = 49) and 13.6% (*n* = 20) in the Moderate and High function clusters, respectively. Finally, our logistic regression results showed that cluster membership was significantly associated with PGIC responses (χ² = 7.82, *p* < 0.02). Specifically, we found that cluster membership in the high-function cluster was associated with higher odds of a positive response compared with the low-function cluster (OR = 1.36, 95% CI 1.06–1.74), whereas no association was observed for the moderate-function cluster.

## Discussion

In this study, we examined the identification of novel subgroups or clusters using only spinal motion data captured from wearable sensor-enabled functional assessments. Our results identified 3 distinct motion clusters with varying degrees of functional impairment. These clusters consisted of: (1) Poor function cluster that consisted of older, obese patients with suboptimal spinal kinematics and moderate to severe pain intensity; (2) Moderate function cluster that had patients with moderate spinal kinematics and mild to moderate pain intensity; and (3) Patients in High function cluster were relatively younger with lower BMI than other clusters, and demonstrated preserved or relatively normal spinal kinematics alongside mild to moderate pain intensity. Interestingly, when assessing differences between clusters, we found that anxiety, depression, sleep disturbance, pain catastrophizing and fear avoidance were biopsychosocial measures that did not have significant variation between clusters, whereas measures (such as pain intensity, pain interference, physical function, social role, fatigue, and self-efficacy) had significant differences. Furthermore, we found a significant association between these motion-based clusters and longitudinal responses (improvement, no change or worsening). Overall, these findings suggest that sensor-based spinal motion assessments have a unique potential to provide objective context on disease severity and enable the identification of novel phenotypes of LBP to enhance patient selection and refine treatment strategies.

An innovative aspect of our study was the use of standardized, in-clinic sensor-based spine-specific motion assessments to identify kinematic clusters. This approach aligns with the theoretical premise that spinal motion signatures offer an indirect objective window into the neuromusculoskeletal system’s response to LBP. It also taps into the use of “spinal function”, a critical domain that is highly valued by both patients and clinicians as a meaningful measure of quality of life. These motion signatures reflect a patient’s motor control pattern which can be altered due to their unique pain experience and is often very distinct from the motion signature of asymptomatic individuals [21, 22]. Our findings suggest that spinal motion features extracted from positions, velocities and acceleration signals can serve as objective indicators for use in the identification of unique subgroups of patients with LBP as evident in our current study. While previous studies have employed various clustering techniques on different biopsychosocial variables, none have specifically targeted spine-specific motion signatures to identify phenotypic variants and their association to critical biopsychosocial domains. From a clinical perspective, these clusters showed distinct variation in severity with significant association to relevant biopsychosocial outcomes. Furthermore, we see that these phenotypes or clusters derived from motion hold considerable potential to guide and tailor treatments especially movement-oriented therapies such as PT, Yoga, or Pilates to improve outcomes for patients with LBP. For example, individuals within a low-function cluster may benefit from interventions focused on restoring mobility and movement capacity, whereas those in a high-function cluster may be better served by pain education, self-management strategies, and behavioral approaches to support long-term condition management. Importantly, prospective studies are needed to determine whether phenotype-informed treatment matching leads to improved outcomes compared with usual care. Numerous studies have explored the identification of pain phenotypes using a wide range of measures, including self-reported biopsychosocial questionnaires, biomechanical assessments, sensory profiles, and imaging features [31, 53–55]. In a recent study by Wilson et al. (2023), psychological factors such as somatization, depression and anxiety, along with sensory profiles measured via pressure pain threshold, were used to identify three distinct clusters [31]. However, they did not observe a significant association between their clusters and longitudinal outcomes of pain ratings and disability at 4 weeks. Similarly, Mohanty et al. (2024) identified 4 prognostic phenotypes in patients with spinal deformity using machine learning algorithms that clustered patients based on their baseline patient reported outcomes (PROs), Edmonton frailty, age, surgical history, and overall health to predict surgical outcomes [53]. Researchers have also discovered similar observations in other chronic pain conditions such as knee osteoarthritis. For instance, Neelapala et al. (2024) found 4 distinct pain phenotypes in patients with early-stage knee osteoarthritis using sensory, psychosocial, behavioral, biomechanical and magnetic resonance imaging features, though no significant link was found between these phenotypes and worsening pain [54]. Finally, in examining the relationship between cluster membership and general measures of pain-related fear-avoidance beliefs, our results were consistent with those of Matheve et al. (2019) [56]. Specifically, they also reported no significant association between lumbar range of motion and general fear avoidance beliefs, but rather task-specific measures of pain-related fear showed stronger associations with movement.

Objective and clinically actionable measures are currently lacking in the management of LBP to better inform treatment selection to optimize outcomes. While PROs are widely used in clinical practice to evaluate key biopsychosocial domains of spine health, they are inherently subjective, relying on patient perceptions that can be influenced by several factors such as cultural, psychological, social etc. This subjectivity can provide an incomplete picture of disease progression and undermine clinical decision-making. These motion clusters are mechanistic, capture intrinsic compensatory movement strategies and reflect variation in actual spine-specific functional impairment rather than perceived function. As such, spinal motion assessments have the potential to serve as meaningful primary endpoints and enhance evaluation of treatment effectiveness by contributing a measure independent of a patient’s perceived subjective status. Therefore, by incorporating objective, sensor-based assessments alongside PROs, there is potential to enhance patient phenotyping and inform more tailored treatment strategies, ultimately improving outcomes. Our results further demonstrate the ability of these sensor-based motion assessments to identify distinct clusters, showing significant association with other biopsychosocial domains (e.g. pain intensity, pain interference, physical function, social role and self-efficacy).

### Strengths and limitations

Strengths of this study include its large sample size and the use of objective, quantifiable kinematic measures from sensor-based functional assessments to address key gaps in understanding the heterogeneity of LBP. This identification of novel phenotypic clusters in a relatively large cohort has the potential to further inform tailoring of patients to the right treatment to improve outcomes. Future efforts can focus on determining the best treatment strategy for each cluster to enhance clinical translation of these findings. Our study also has several limitations. First, our study was conducted at a single tertiary institution located in midwestern U.S, which could impact generalizability of our findings. Second, while unintentional, our cohort had a higher proportion of females than males. Third, the phenotypes derived in this effort were not externally validated and further studies are needed. Fourth, this was an observational study on LBP population that received a wide variety of treatments, which can impact generalizability of our findings. Lastly, we analyzed longitudinal outcome at 3 months only and so this study could benefit from more longer-term follow-up data.

## Conclusion

Our study identified 3 distinct motion-based clusters that showed significant association with relevant biopsychosocial factors of LBP. Our results further support the novel use of sensor-based spinal motion assessments for phenotyping of LBP and highlights between cluster differences at baseline across many biopsychosocial measures as well as variation in longitudinal responses between clusters. Finally, our study suggests that sensor-based assessments can provide a means of objectively evaluating the extent of functional impairment and indirectly capture intrinsic biopsychosocial factors that are affected by LBP to better inform clinical decision-making on disease severity and treatment selection.

## Data Availability

All relevant data may be made available upon request.
